# PEGylated Liposomes Remotely Loaded with the Combination of Doxorubicin, Quinine, and Indocyanine Green Enable Successful Treatment of Multidrug-Resistant Tumors

**DOI:** 10.3390/pharmaceutics13122181

**Published:** 2021-12-17

**Authors:** Emma Grabarnick (Portnoy), Alexander V. Andriyanov, Hadas Han, Sara Eyal, Yechezkel Barenholz

**Affiliations:** 1 Department of Biochemistry, Institute for Medical Research Israel-Canada, Hadassah Medical School, Hebrew University, P.O. Box 12272, Jerusalem 9112102, Israel; grabarne@mskcc.org (E.G.); alex.andri@mail.huji.ac.il (A.V.A.); 2 Institute for Drug Research, Faculty of Medicine, Hebrew University, Jerusalem 9112102, Israel; hadashan@mail.tau.ac.il (H.H.); sarae@ekmd.huji.ac.il (S.E.)

**Keywords:** liposomes, ICG, phototherapy, multi drug resistance

## Abstract

Multidrug resistance (MDR) of cancer cells remains a major obstacle to favorable outcomes of treatment with many drugs, including doxorubicin. Most of the clinical trials failed to demonstrate the benefit of the drug efflux transporter P-glycoprotein (P-gp) inhibitors to circumvent P-gp-mediated drug resistance in vivo. The present study explored the therapeutic potential of combined treatment with liposomal doxorubicin, P-gp inhibitor quinine, and the photodynamic therapy (PDT) using indocyanine green (ICG) in the adenocarcinoma drug-resistant tumor model. Liposomes were actively co-remotely loaded with doxorubicin and quinine, and ICG was passively adsorbed. The liposomes were characterized by differential scanning calorimetry (DSC) and cryogenic transmission microscopy (Cryo-TEM). We found that quinine impaired the crystalline structure of doxorubicin. In vitro, treatment with single agents themselves was insufficient to inhibit the growth of HT-29 MDR1 cells. However, pegylated liposomal doxorubicin and quinine (PLDQ) significantly diminished HT-29 MDR1 cell survival. Furthermore, survival inhibition intensified by the addition of ICG to the PLDQ (ICG + PLDQ). In vivo, ICG + PLDQ significantly decreased tumor growth when combined with tumor irradiation with NIR light (** *p* < 0.01). ICG + PLDQ + irradiation was superior to single treatments or combinational treatments without irradiation. These findings suggest that ICG + PLDQ can overcome P-gp-mediated MDR in cancer cells.

## 1. Introduction

Despite advances in chemotherapies against cancer, multidrug resistance (MDR) still hampers positive therapeutic outcomes [1]. MDR, characterized by cross-resistance to multiple functionally and structurally unrelated drugs [2], often caused by the treatment [3] but may also be intrinsic to many tumor types. One established mechanism of MDR is P-glycoprotein (P-gp/ABCB1), a member of the ATP-binding cassette superfamily of transporters (ABC). P-gp is expressed in many cancer types [4], and its presence correlates with poor prognosis [5,6,7]. Among P-gp substrates, there is a broad range of medications, including anti-cancer drugs such as doxorubicin and paclitaxel [8,9]. The usage of P-gp inhibitors such as cyclosporin A and quinine in addition to chemotherapeutic protocols resulted in more extended relapse-free periods and better overall survival [10,11,12,13]. However, drug-drug interactions, toxicity, and design have led to the failure of most clinical trials [13,14].

One strategy for building up effective concentrations of chemotherapeutic agents in tumor cells is incorporating them within liposomes. PEGylated, long-circulating nanoliposomes such as liposomal Doxorubicin (Doxil^®^) [15] predominantly accumulate in tumor cells [16]. The tumor accumulation of such liposomal drugs is mediated by the enhanced permeability and retention (EPR) effect caused by the leaky tumor vasculature and poor lymphatic drainage [17,18]. The release of doxorubicin at the tumor site is induced by the high concentration of ammonium/ammonia generated in the tumor by glutaminolysis (cancer cells’ unique metabolic pathway) [19,20,21,22]. Nano-range size and PEGylation reduce the uptake of these liposomes by the reticuloendothelial system (RES), thus increasing their circulation time [16] and eventually improving the probability of successful therapy with reduced drug toxicity. Unfortunately, in MDR-resistant malignancies, the drug delivered by liposomes is still excluded from tumor cells [23]. Therefore, different approaches are required for overcoming MDR.

To further improve the therapeutic efficacy of liposomal doxorubicin, we included in the same PEGylated nano-liposomes an FDA-approved photosensitizer (Indocyanine green, ICG) to enable combining photodynamic therapy (PDT) and chemotherapy. ICG is superior to most other photosensitizers due to its better tissue penetration and good safety profile. Delivery of liposomal ICG to the tumors due to the EPR effect [24] and its exposure to near-infrared (NIR) light produces reactive oxygen species that act against the tumor cells [25,26]. Because doxorubicin and ICG are P-gp substrates, we further added quinine to the liposomes, an established P-gp inhibitor [27]. Here, we compared the antitumor efficacy of doxorubicin and quinine liposomes to that of combined phototherapy involving adsorbed ICG both in vitro and in vivo.

## 2. Materials and Methods

### 2.1. Materials

A mixture of hydrogenated soy phosphatidylcholine (HSPC), cholesterol *N*-carbamyl-poly-(ethylene glycol methyl ether)-1,2-distearoyl-sn-glycero-3-phosphoethanolamine sodium salt (PEG2k-DSPE) was obtained from Lipoid (Ludwigshafen, Germany). Doxorubicin hydrochloride was obtained from Teva (Tenuta, Italy). Indocyanine green (ICG) was from Acros (Geel, Belgium). Quinine and all other reagents were purchased from Sigma-Aldrich (Rehovot, Israel). All cell culture reagents and the 2,3-bis-(2-methoxy-4-nitro-5-sulfophenyl)-2H-tetrazolium-5-carboxamide, (XTT) colorimetric viability assay were from Biological Industries (Beit Ha Emek, Israel).

### 2.2. Preparation of Liposomal Drug for the In Vitro and In Vivo Studies

PEGylated nano-liposomes of similar size distribution and lipid composition to Doxil^®^ were produced from a lipid mixture of HSPC- cholesterol-PEG-DSPE, in a mole ratio 57:38:5 (weight ratio of 3; 1; 1), using the procedure described elsewhere [28,29].

The lipids were hydrated with an ammonium sulfate buffer at a concentration of 500 mM in Doxil in order to improve the co-remotely loading of doxorubicin plus quinine. The total lipid concentration was 40 mg/mL. The final doxorubicin and quinine concentrations were 2 mg/mL and 1 mg/mL, respectively. Doxorubicin concentration was determined as previously described [30], and that of quinine by a colorimetric assay [31]. The formulation of PEGylated nano-liposomes co-loaded with doxorubicin and quinine is referred to as PLDQ.

### 2.3. Incorporating ICG into PLD and PLDQ

Aliquots of 6 mM ICG in aqueous solutions were added to PEGylated Liposomal Doxorubicin (PLD) and PEGylated Liposomal Doxorubicin quinine (PLDQ), to a final ICG concentration of 1 mM. This dispersion was mixed for 1 h at 4 °C before the use of liposomes in vivo or in vitro.

### 2.4. Differential Scanning Calorimetry (DSC) Measurements

All liposomal preparations were analyzed using a high-sensitivity differential scanning calorimeter MicroCal VP-DSC system (Malvern, Worcestershire, UK). The scanning was performed at the rate of 1 °C/min, according to previous studies [29]. Prescan, the samples were degassed by the MicroCal ThermoVac system. The sample and the reference were scanned in a few cycles of heating-cooling-reheating. MicroCal LLC DSC workstation software (Malvern, Worcestershire, UK) was used to calculate parameters according to previous publications [32].

### 2.5. Cell Culture

P-gp overexpression was induced in HT-29 cells by the gradual increase of colchicine concentration in the growth medium. The final concentration of colchicine was 300 ng/mL. Both control HT-29 cells and these overexpressing P-gp (HT-29 MDR1) were grown in Dulbecco’s modified Eagle’s phenol-free low-glucose medium (DMEM). The medium was supplemented with 10% fetal bovine serum, 2 mM L-glutamine, 100 units/mL penicillin, and 100 μg/mL streptomycin at 37 °C in a 5% CO_2_ incubator.

### 2.6. In-Vitro Assays

For toxicity studies, 20,000 cells per well were seeded in 96 wells plates. Twenty-four hours post-seeding, cells were co-incubated with liposomal preparations. PDT has carried out 24 h post-incubation by exposing the cells for 10 min to 780 nm, 55 mW irradiation. At the end of irradiation, cells were returned to the incubator for 30 min. Then, the medium was replaced. The XTT colorimetric assay was used to determine cell viability.

### 2.7. Western Blot Analysis

The cells were washed with ice cold PBS for western blot analysis and lysed by 200 µL of lysis buffer (0.01 M Tris-HCl, pH 7.5, 0.1% sodium dodecyl sulfate (SDS), 0.01 M MgCl_2_ and protease inhibitors cocktail). The proteins were extracted by mixing cells for one hour at 5 °C and consequent centrifugation for 15 min at 14,300 rpm. A BCA Protein Assay Reagent Kit (Pierce, Rockford, IL, USA) was used for the determination of the protein amount. The gels were electrotransferred to nitrocellulose membranes. The membrane was blocked by 0.1% TBST (Tris-buffered saline) and 5% milk powder and incubated overnight at 4 °C with specific anti-P-gp antibodies (C219, 1:1000) and β-actin antibody (1:2500, both from Biotest (Kfar Saba, Israel). The peroxidase-conjugated goat anti-rabbit secondary antibody or goat anti-mouse IgG (1:10,000) were incubated with the membrane for one hour at room temperature.

Cells were washed twice with cold PBS. Whole-cell lysates were prepared in an ice cold lysis buffer containing 200 µL of 0.01 M Tris-HCl, pH 7.5, 0.1% sodium dodecyl sulfate (SDS), 0.01 M MgCl_2_, protease inhibitors cocktail. The cells were shaken with the lysis buffer for one hour at 5 °C. The lysate was centrifuged for 15 min at 14,300 rpm. Protein concentrations were determined by the 54 BCA Protein Assay Reagent Kit (Pierce, Rockford, IL, USA). Following SDS-PAGE analysis under reducing conditions, gels were electrotransferred to nitrocellulose membranes. Membranes were blocked in Tris-buffered saline containing TBST (0.1%) and milk powder (5%) and probed overnight at 4 °C with specific antibodies C219 at 1:1000 and β-actin antibody at 1:2500. The blots were then incubated with peroxidase-conjugated goat anti-rabbit secondary antibody or goat anti-mouse IgG at 1:10,000 for 1 h and developed by enhanced chemiluminescence.

### 2.8. The In Vivo Animal Models

The procedures described in the current article were conducted in accordance with protocols approved by the Animal Care and Use Committee of the Hebrew University (Protocol # MD-17-15156-4, approval date 16 May 2017). The experiments were performed with male seven to eight week old CD1 nude mice obtained from Harlan Laboratories (Rehovot, Israel). The mice were maintained in SPF (specific pathogen-free facility) on an automatically timed light/dark cycle and had free access to food.

HT29-MDR1 cells were injected into the right and left flanks (five million cells) to assess the added value of PDT within the same mice. Direct caliper measurements were utilized for the determination of tumor size [33]. Four to six weeks later, when the tumors grew to the desired diameter, 8 ± 2 mm, the 28 tumor-bearing mice were randomized to five groups: (1) PLDQ-ICG; (2) Lip-ICG; (3) PLD-ICG; (4) PLD (5) untreated, control mice. The total dose of doxorubicin given to each mouse was 8 mg/kg [34]. One of two tumors was treated by PDT, using 55 mW NIR light (780 nm, for 10 min) two hours post-injection of liposomal preparations. The PDT procedure was performed two hours after the drug injection. Thus, the mice were treated four times, with five days between treatments.

The two primary endpoints were the tumor growth rate and the time to achieve a surrogate endpoint of tumor burden (the burden of tumor more than 1000 mg, about four-fold tumor growth, which was considered the humane endpoint). The endpoints and body weight were monitored two or three times per week.

Tumor weights were calculated according to the equation:

$$\mathrm{Tumor weight}\;\left(g\right)=\frac{\mathrm{length}\times {\mathrm{width}}^{2}}{2}$$

After the last treatment, tumor size was measured again by a caliper, and then mice were sacrificed by cervical dislocation. Mice were anesthetized by isoflurane (1–2% *v*/*v*) before imaging by IVIS (Caliper).

### 2.9. Statistical Analysis

The statistical significance of the difference between treatment groups was determined using a 2-way ANOVA test for tumor volume and the Log-rank (Mantel-Cox) test for comparison of Kaplan-Meir curves (Mantel 1966). A *p* value of less than 0.05 was considered significant. The statistical analysis was performed using Prism 9.02 software (GraphPad, San Diego, CA, USA).

## 3. Results

### 3.1. Characterization of Liposomal PLD and PLDQ Formulations

**Figure 1:**
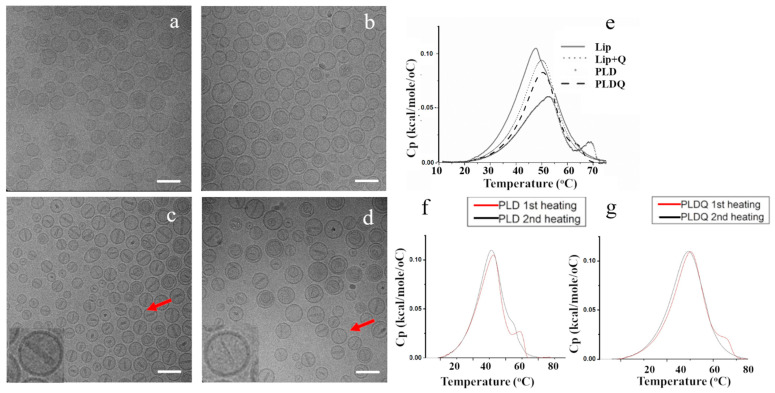
Structural characterization of PLD and PLDQ. (**a**). Cryo-TEM images of empty liposomes (Lip), liposomes remotely loaded with quinine only, (**b**). (Lip + Q); (**c**). liposomal doxorubicin(PLD), (**d**). liposomal doxorubicin, and quinine (PLDQ). The insert is sixfold digital magnification. Scale bar 100 nm. (**e**). DSC thermograms for various liposomal preparations. (**f**). DSC thermograms after two heating cycles of PLD. (**g**). DSC thermograms after two heating cycles of PLDQ.

We compared and characterized different formulations by Cryo-TEM and DSC (Figure 1). Cryo-TEM revealed (Figure 1b) that no crystal was formed in the intra-liposomes of liposomes remotely loaded with quinine alone (Lip + Q) and with DSC thermograms (Figure 1e). The quinine solely increased lipid Tm value compared to that of empty liposomes (Lip) 48.1 ± 0.4 °C to 50.0 ± 0.2 °C (Figure 1e, Table S1), suggesting that at least a fraction of the quinine is interacting with the liposomal membrane. Quinine compromised the crystalline structure of doxorubicin sulfate at the intra-liposomes aqueous phase in PLDQ. Compared to doxorubicin-only liposomes (PLD), there was a visible decrease in the size of doxorubicin crystals in PLDQ, and their structure was less defined (Figure 1d). DSC thermograms demonstrated a reduction in the melting point of the doxorubicin-sulfate crystals from 68.6 ±0.8 to 64.1 ±0.5 °C (Figure 1f,g, Table S1). During the second heating cycle, the doxorubicin peak was less pronounced, yet still visible for PLD, while it completely disappeared for PLDQ. We measured the release rate of doxorubicin and quinine from the liposomes using ammonium sulfate in histidine buffer (50 mM and 10 mM, pH = 7.4, respectively) to induce the release [32,35]. As expected, the release for both doxorubicin and quinine was induced by ammonium (Figure S1), while for doxorubicin, the release increased from 11% (at 4 °C) to 52% at 37 °C. For quinine, the release increased from 40% (at 4 °C) to 60% at 37 °C.

Loading with quinine didn’t influence the size and zeta potential of liposomes. The size of PLD liposomes measured by dynamic light scattering (DLS) was 100.8 ± 2.1 nm, and for PLDQ, 105 ± 2.9 nm and zeta potential −7.9 ± 0.57 mV and −7.7 ± 0.37 mV.

### 3.2. Characterization of HT-29 MDR Cells

**Figure 2:**
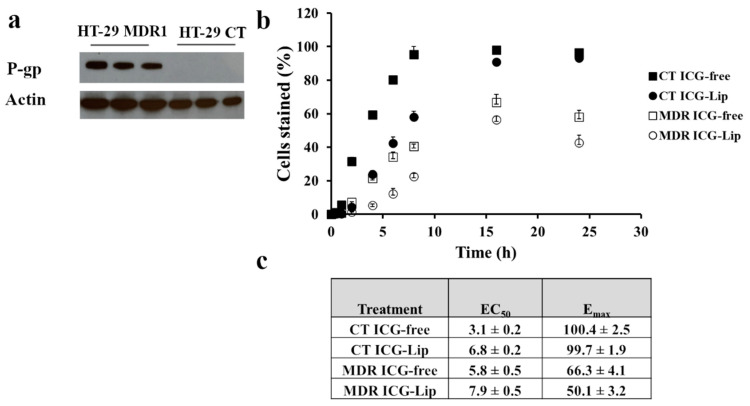
Characterization of P-gp in HT-29 CT and HT-29 MDR1 cell lines. (**a**). Western blot analysis of P-gp expression in HT-29 CT and HT-29 MDR1 cell lines. β-actin was used as the reference (**b**). FCS analysis of free ICG uptake or Lip-ICG, (**c**). EC50 and E_max_ values derived from uptake profile of free ICG or Lip-ICG in HT-29 CT and HT-29 MDR1 cells, *n* = 3. Full and open markers represent non-irradiated and irradiated cells, respectively. Values represent mean ± SD.

P-gp overexpression in HT-29 MDR1 cells was confirmed by western blotting (Figure 2a, Figure S5). We confirmed P-gp functionality by measuring ICG uptake in HT-29 cells [36,37]. The uptake of free ICG was higher for HT-29-CT cells compared to HT-29 MDR1 as indicated by lower EC_50_ values of cell labeling (3.1 ± 0.2 min vs. 5.8 ± 0.5 min, Figure 2b,c) and higher E_max_ (100.4 ± 2.5 vs. 66.3 ± 4.1%, respectively; Figure 2c). ICG adsorption to liposomes (Lip-ICG) did not protect it against P-gp-mediated efflux as indicated by similar E_max_ values for free ICG from HT-29 MDR cells vs. Lip-ICG (66.3 ± 4.1 vs. 50.1 ± 3.2, Figure 2c). ICG accumulation rate was slower in liposomes as indicated by higher EC_50_ values for liposomes compared to free form (7.9 ± 0.5 min vs. 5.8 ± 0.5 min for HT-29 MDR1 cells and 6.8 ± 0.2 min vs. 3.1 ± 0.2 min, for HT-29 CT cells, Figure 2c).

### 3.3. Cytotoxicity of Free and Liposomal Drugs in HT-29 CT vs. HT-29 MDR1 Cells

**Figure 3:**
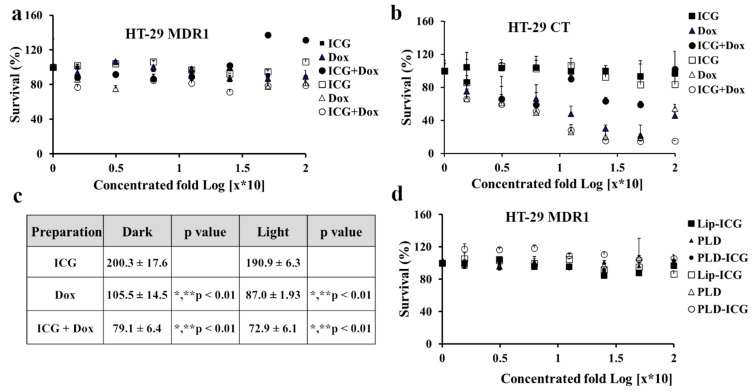
Toxicity of doxorubicin and ICG in HT-29 CT and HT-29 MDR1 cells with and without NIR irradiation. (**a**). Survival (%) HT-29 MDR1 cells treated by free ICG and doxorubicin. (**b**). Survival (%) HT-29 CT cells treated by free ICG or doxorubicin. (**c**). Analysis of area under the curve (AUC) of toxicity curves in HT-29 CT cells, based on Figure 2b. (**d**). Survival (%) HT-29 MDR1 cells treated by liposomal ICG and doxorubicin. % Survival normalized to control untreated cells. 2-way ANOVA Tukey Multiple Comparisons Test *n* = 6. The filled and open markers represent non-irradiated and irradiated cells, respectively. * compared to ICG, not irradiated, ** compared to ICG, irradiated. Values represent mean ± SD.

Next, we evaluated ICG and doxorubicin cytotoxicity either as monotherapies or in combination. HT-29 cells were irradiated by 780 nm NIR led (Figure 3a). Doxorubicin alone or in combination with ICG was not toxic to HT-29 MDR1 cells (Figure 3a). At the same time, HT-29 CT cell viability was significantly decreased in the presence of doxorubicin (*p* < 0.01, Figure 3b,c). In addition, the cell growth was lower in combination with ICG (compared to *p* < 0.01, Figure 3b,d). Interestingly, free ICG alone, either irradiated or non-irradiated, did not influence viability in HT-29 CT cells (Figure 3b). Corresponding liposomal drugs were also not toxic for HT-29 MDR1 cells, either when irradiated or not irradiated (Figure 3d).

### 3.4. Cytotoxicity Measurements of Free and Liposomal Drugs in HT-29 MDR1 Cells in the Presence of P-Gp Inhibitor Quinine

**Figure 4:**
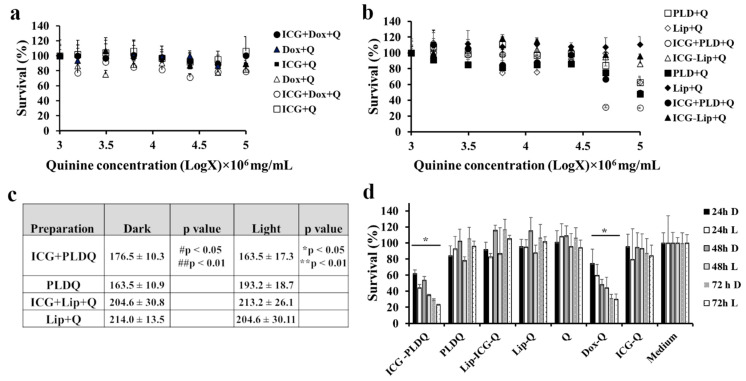
Effect of quinine, a P-gp inhibitor, on the survival of HT-29 MDR1 cells. (**a**). Toxicity of free doxorubicin, ICG, or combination of free drugs in HT-29 MDR1 cells. (**b**). Toxicity of liposomal doxorubicin, ICG, quinine, or combination of drugs in HT-29 MDR1 cells. (**c**). AUC of toxicity curves in HT-29 CT cells, based on Figure 2b, * *p* < 0.05 compared to ICG + PLDQ non-irradiated, PLDQ irradiated, ** *p* < 0.01 compared ICG-Lip + Q non-irradiated, Lip + Q non-irradiated/irradiated, # *p* < 0.05 compared to Lip + Q light, ## *p* < 0.01 compared to ICG-Lip + Q non-irradiated, Lip + Q non-irradiated. (**d**). Survival of HT-29 MDR1 cells in the presence of liposomal drugs or free drugs after 24, 48, and 72 h. D- not irradiated, L-irradiated, * *p* < 0.01 compared to the medium, *n* = 6/group; ANOVA with post-hoc Tukey Multiple Comparison Test. The filled and open markers represent non-irradiated and irradiated cells, respectively. Values represent mean ± SD.

Free quinine, doxorubicin, and ICG were not toxic to either in irradiated or non-irradiated form for free drugs (~20% compared to untreated cells, Figure 4a). At the same time, the liposomal formulation of quinine, doxorubicin, and ICG (ICG + PLDQ) resulted in a significant reduction in cell viability (Figure 4b,c). The maximal effect achieved for irradiated cells treated by PLDQ was 30.4 ± 0.4 and 49.4 ± 2.9% without irradiation (Figure 4b,c). We measured viability as a function of time (Figure 4d). The more prolonged exposure of ICG + PLDQ and PLDQ further induced cytotoxicity for each tested concentration and was significantly higher than medium (** *p* < 0.01), with maximal effect after 72 h. All other formulations were not toxic to cancer cells.

### 3.5. In Vivo Efficacy Study

**Figure 5:**
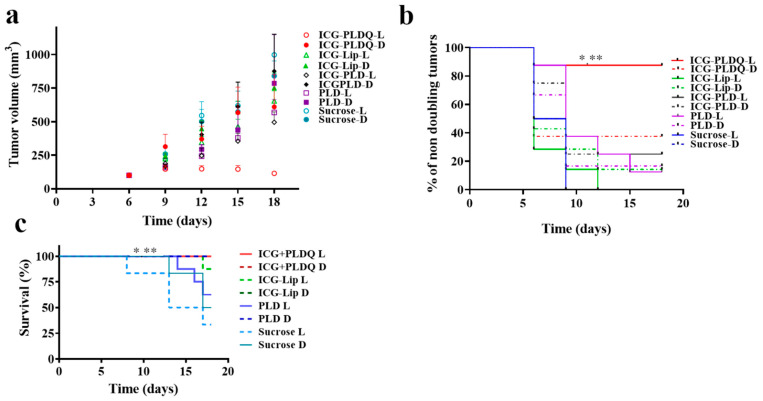
Tumor progression: (**a**). The tumor weight changed as a function of time. Values represent mean ±SE. (**b**). Percent of non-doubling tumors. Long rank test (Mantel Cox) * *p* < 0.05 compared to PLDQ-D, ICG-PLD D/L, PLD D, ** *p* < 0.01 compared to Sucrose L, Sucrose D, *n* = 6–8. (**c**) Survival plot for exclusion of mice from the experiment. Long rank test (Mantel-Cox) ** *p* < 0.01 significantly different from sucrose L, * *p* < 0.05 significantly different from sucrose D.

We characterized the anti-resistant tumor efficacy of various liposomal formulations in HT-29 MDR1 mice xenograft in-vivo (Figure 5a,b). The tumor growth rate as evaluated by tumor volume was significantly lower for ICG + PLDQ L compared to both sucrose treated groups (** *p* < 0.01), PLD D (* *p* < 0.05, Figure 5a, Figure S2). The results were supported by ex-vivo measurement of doxorubicin fluorescence emission intensity of tumors treated by ICG + PLDQ and ICG-PLD. In addition, we found that accumulation was higher in the ICG + PLDQ group (Figure S3).

The mice tumor doubling time was significantly lower for the ICG + PLDQ group exposed to light (ICG + PLDQ L), * *p* < 0.05 compared to non-irradiated (ICG + PLDQ D) or sucrose irradiated groups (sucrose L, Figure 5b). Moreover, the mouse survival (mice were excluded based on the tumor size and condition) was significantly lower for ICG + PLDQ in the irradiated and non-irradiated treatment groups than the sucrose-treated groups (Figure 5c).

## 4. Discussion

In the current study, we combined quinine and doxorubicin in one liposome to produce a maximally efficacious dose in P-gp overexpressing tumors. This combination resulted in a synergy that may be explained by inhibition of the P-gp pump and additional effects such as accumulation of reactive oxygen species and more rapid release of doxorubicin from liposomes. Based on cryo-TEM and DSC data (Figure 1), quinine was remotely and actively loaded into the intraliposomal aqueous phase. Doxorubicin and quinine are weak bases and, therefore, may be loaded by a remote loading mechanism driven by an ammonium sulfate gradient [38]. Quinine seems to disturb the intra-liposome nanorod crystals of doxorubicin-sulfate. Based on previous studies [32], such changes in the physical state of the intra-liposome doxorubicin crystal may result in a much faster release rate from the liposomes. In addition, quinine may promote the release of doxorubicin from the lysosomal compartment to the cytosol by elevating endosomes/lysosomes pH similarly to the effect of chloroquine [39,40].

Moreover, previous studies demonstrated anti-cancer properties of quinine independent of P-gp inhibition [41]. Structurally, doxorubicin in PLDQ liposomes is different from classical Doxil bundles [42] (Figure 1). Quinine interferes with the crystalline doxorubicin structure since the rods seem to be thinner and have lower intensity than in PLD (Figure 1a–d). DSC thermograms support Cryo-TEM data; the characteristic peak of doxorubicin is less sharp and has lower Tm than in PLD, both effects support the image of less dense crystal, which, as discussed above, may explain the faster release of doxorubicin from PLDQ than from PLD. Contrary to PLD, for PLDQ liposomes, the typical peak of doxorubicin disappears after the second cycle of heating (Figure 1e–g, Table S1).

We further characterized HT-29 MDR1 as a model for resistant colon cancer. As expected, we observed a higher accumulation of ICG, P-gp substrate [37] in HT-29 CT compared to P-gp overexpressing HT-29 MDR1 cells. (Figure 2). Liposomal ICG was prone to efflux by P-gp similarly to free ICG, as indicated by similar Emax values (Figure 2b,c). Liposome encapsulation probably affected the release of ICG since EC50 for liposomal ICG was almost twice as low.

Contrary to previous studies on cancer cells, irradiation of either free ICG or encapsulated [25,43] neither influenced the growth of HT-29 control nor MDR1(Figure 3a,b). However, we observed a heating effect (up to 10 °C, Figure S4a), and radical production (Figure S4b). Free doxorubicin, the known substrate of P-gp [44], as expected, did not affect the growth rate of HT-29 MDR1 cells while significantly inhibiting the growth of HT-29 CT cells (Figure 3b,c). Interestingly, a combination of ICG with doxorubicin resulted in higher toxicity of HT-29 CT compared to both free drugs, either irradiated or not irradiated (Figure 3b,c). The effect may be explained by the higher radical species load produced by doxorubicin [45] and ICG [46]. Besides the DNA intercalating mechanism, doxorubicin can generate a variety of free radical species in cells [45].

Contrary to some previous reports [47,48], and in agreement with other publications [49,50], liposomal doxorubicin (PLD) did not circumvent P-gp efflux. In the current study, the liposomal doxorubicin was not toxic to HT-29 MDR1 cells (Figure 4b), similarly to free doxorubicin (Figure 3a). It was suggested in the literature that in vivo liposomal doxorubicin improved the outcome of resistant tumors due to modified pharmacokinetics parameters and the high bioavailability of the liposomal doxorubicin [51]. However, the HT-29 MDR1 resistant cells viability was decreased only in the presence of P-gp inhibitor quinine (Figure 4d). Furthermore, we observed potent inhibition for the PEGylated nano-liposomes co-remotely loaded with the combination of doxorubicin and quinine (Figure 4b–d). Therefore, we anticipate that co-administration of all drugs in one liposome will be advantageous for delivering all components at once to the same tumor site. This assumption was supported by a lack of efficiency following incubation of free drugs with quinine (Figure 4a). A combination of doxorubicin and quinine in liposomes was reported previously and was sufficient to inhibit P-gp in the MCF-7 cell line in vitro [50]. However, liposomal PLDQ was less efficient than ICG + PLDQ in-vitro in HT-29 MDR1 cells (Figure 4). In our study, the addition of ICG to PLDQ demonstrated a synergistic effect both in vitro and in vivo ( Figure 4 and Figure 5). However, in vivo, a significant improvement of survival and tumor volume reduction was achieved by the addition of irradiation on ICG + PLDQ (Figure 5). Most likely, irradiating ICG produces reactive oxygen species and local heating [46], while the liposomal formulation augments these effects (Figure S4).

## 5. Limitations of the Study

Since the cancer microenvironment is unique and has a complex, chaotic structure, one mechanism cannot explain resistance, which is likely mediated by multiple processes. Therefore, our current study is limited to the P-gp multidrug-resistant cancer model, and other cancer types should be individually addressed. Moreover, the co-administration of other P-gp modulators may lead to unwanted adverse effects due to drug-drug interactions [13,14,52]. However, we did not observe any toxicity, e.g., adverse effects associated with higher free doxorubicin in circulation, such as excessive weight loss or edema [53]. We believe that this approach has the potential to be utilized clinically for selected drug-resistant groups of cancers.

## 6. Conclusions

Resistance caused by the P-gp transporter is only one of numerous processes responsible for multidrug resistance. Although quinine combined with doxorubicin inhibits proliferating tumor cells in vitro, it is unlikely to be efficacious in vivo, as we demonstrated in the case of the HT-29 MDR1 xenograft model. Photodynamic therapy by liposomal ICG causes a synergistic effect with the chemotherapeutic drug substances doxorubicin and quinine. In our opinion, multiple strategies are needed to overcome resistance due to the lower chances of developing compensatory mechanisms. We anticipate that this technology may treat resistant colon cancer, where visible polyps can be irradiated locally using modern technologies.

## Supplementary Materials

The following are available online at https://www.mdpi.com/article/10.3390/pharmaceutics13122181/s1. Table S1: Melting points measured by DSC expressed as the mean temperature in °C ± STD. Figure S1: Release of Doxorubicin and quinine from liposomes. (a) Release of Doxorubicin from liposomes at 4 °C and 37 °C in histidine (50 mM) and ammonium sulfate (10 mM) buffers. (b) Release of quinine from liposomes at 4 °C and 37 °C. Figure S2: AUC of tumor volume curves. * *p* < 0.05 compared to Sucrose D, PLD-ICG-D, PLDQ-ICGD, ** *p* < 0.01 compared to Sucrose L. *n* = 6–8. Values represent mean ±SE. 2-way ANOVA with Tukey Multiple Comparisons Test. Figure S3: Doxorubicin emission in tumors, ex-vivo. Figure S4: (a) Temperature difference measurement as a function of time. (b) Reactive oxygen species treatment measurement by DCF dye. ** *p* < 0.01. 2-way ANOVA with Tukey Multiple Comparisons Test. Figure S5: Western blot for HT-29 and HT 29 MDR1 cells showing expression of P-gp and beta-actin.
