# Supplementary material for: PEGylated Liposomes Remotely Loaded with the Combination of Doxorubicin, Quinine, and Indocyanine Green Enable Successful Treatment of Multidrug-Resistant Tumors
Source: Pharmaceutics. 2021 Dec 17;13(12):2181. doi: 10.3390/pharmaceutics13122181 (PMC8708987; doi:10.3390/pharmaceutics13122181)
Supplement: Supplementary file 1 [file pharmaceutics-13-02181-s001.zip › pharmaceutics-1485669-supplementary.pdf]

pharmaceutics

MDPI

Supplementary Materials: PEGylated Liposomes Remotely Loaded With the Combination of Doxorubicin, Quinine, and Indocyanine Green Enable Successful Treatment of Multidrug-Resistant Tumors

Emma Grabarnick (Portnoy), Alexander V. Andriyanov , Hadas Han, Sara Eyal and Yechezkel Barenholz

Table S1. Melting points measured by DSC expressed as the mean temperature in °C ± STD.

Liposome Type	Tm 1 (°C)	Tm 2 (°C)
PLD	52.8 ± 0.2	68.6 ± 0.8
PLDQ	50.4 ± 0.1	64.1 ± 0.5
Quinine	50.0 ± 0.2	
Lip	48.1 ± 0.4	

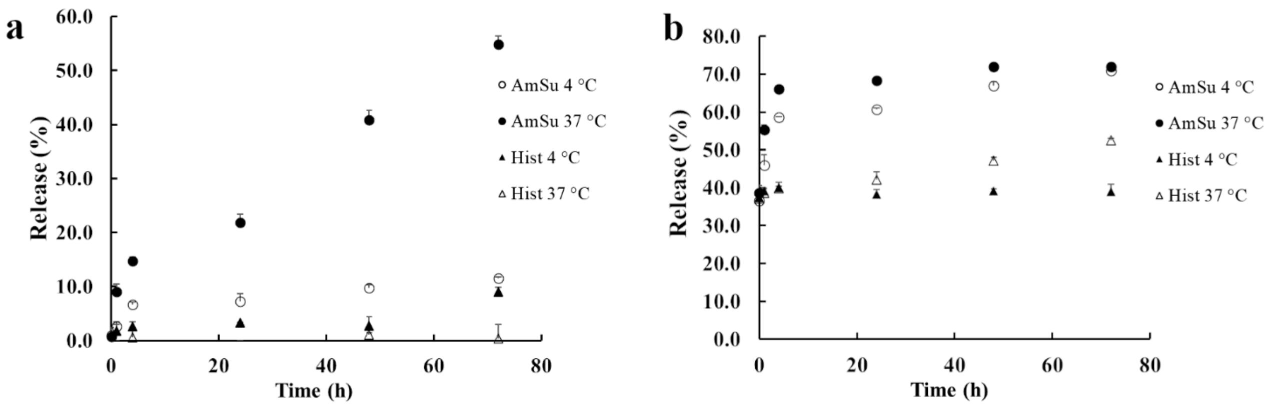

Figure S1. Release of Doxorubicin and quinine from liposomes. (**a**) Release of Doxorubicin from liposomes at 4 °C and 37 °C in histidine (50 mM) and ammonium sulfate (10 mM) buffers. (**b**) Release of quinine from liposomes at 4 °C and 37 °C.

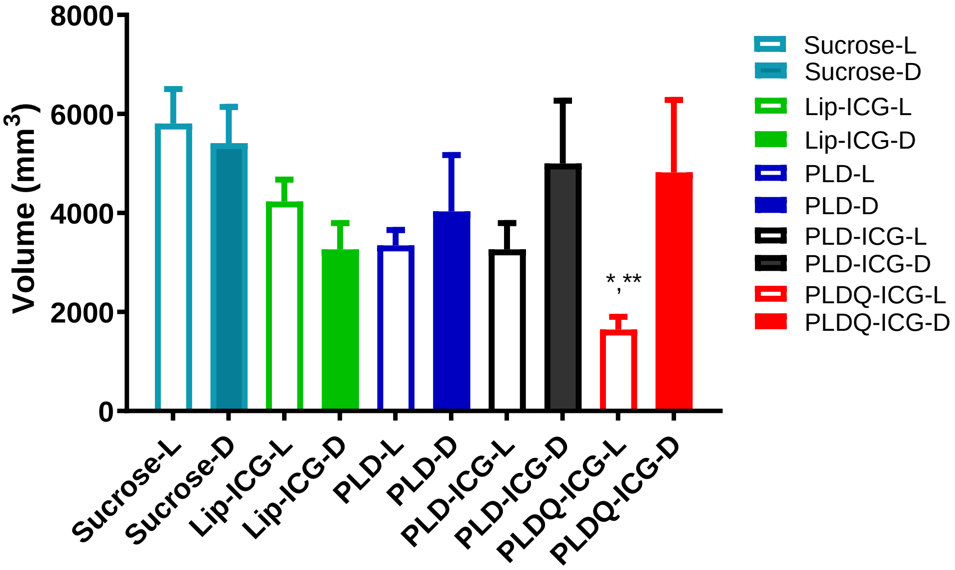

Figure S2. AUC of tumor volume curves. * p <0 .05 compared to Sucrose D, PLD-ICG-D, PLDQ-ICGD, ** $p < 0.01$ compared to Sucrose L. n = 6–8. Values represent mean ±SE. 2-way ANOVA with Tukey Multiple Comparisons Test.

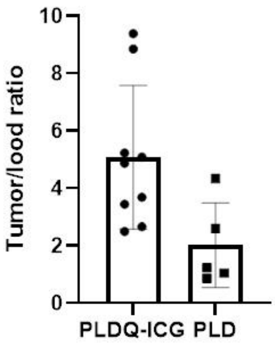

Figure S3. Doxorubicin emission in tumors, ex-vivo.

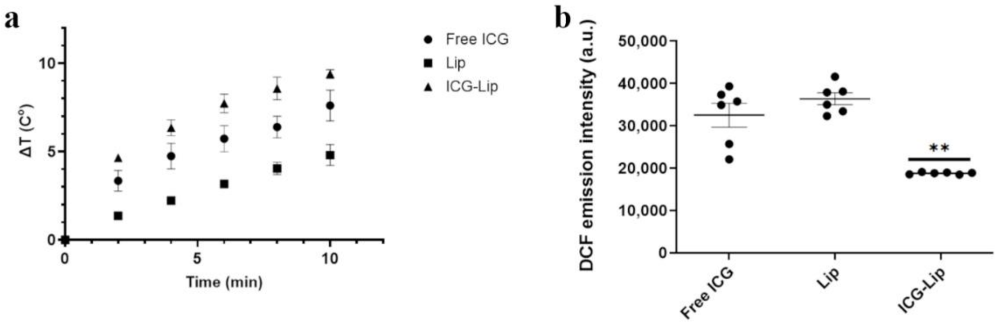

Figure S4. (**a**) Temperature difference measurement as a function of time. (**b**) Reactive oxygen species treatment measurement by DCF dye. ** $p < 0.01$. 2-way ANOVA with Tukey Multiple Comparisons Test.

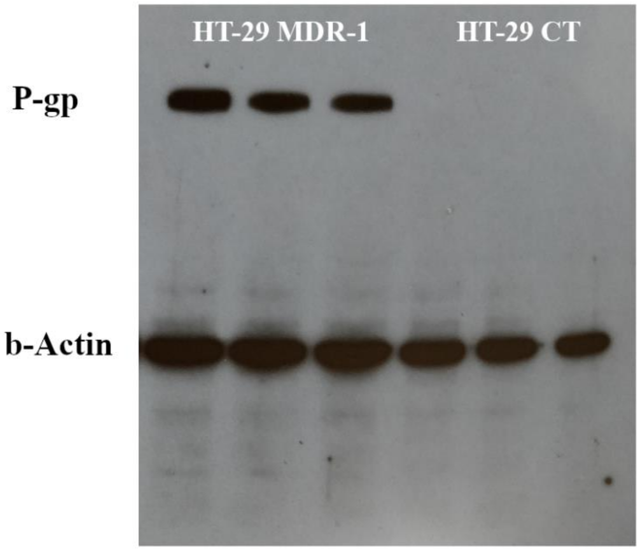

Figure S5. Western blot for HT-29 and HT 29 MDR1 cells showing expression of P-gp and beta-actin.
